# Retained primitive reflexes are independently associated with impaired postural control in adolescents with idiopathic scoliosis

**DOI:** 10.3389/fneur.2026.1921169

**Published:** 2026-09-14

**Authors:** Wenjun Jiang, Peng Zhang, Ningnan Zhu, Jiahuan Wu, Sheng Wang, Xueliang Wu

**Affiliations:** 1 Suzhou Vocational Health College, Suzhou, China; 2 Suzhou Research Center of Medical School, Suzhou Hospital, Affiliated Hospital of Medical School, Nanjing University, Suzhou, China

**Keywords:** adolescent idiopathic scoliosis, balance, computerized dynamic posturography, Galant reflex, neurodevelopment, postural control, primitive reflexes

## Abstract

**Background:**

Adolescents with idiopathic scoliosis (AIS) exhibit impaired postural control, yet the neurodevelopmental factors underlying this dysfunction remain poorly characterized. Postural control in AIS is heterogeneous, and no clinically accessible neurodevelopmental marker currently exists to identify patients with underlying sensorimotor dysfunction independent of age and radiographic curve severity. Primitive reflexes, brainstem-mediated motor responses that are typically integrated in infancy, have been linked to motor deficits in other neurodevelopmental conditions but have not been investigated in AIS. We tested whether retained primitive reflexes are independently associated with postural control impairment in AIS, after controlling for age and curve severity.

**Methods:**

In this cross-sectional study, 48 adolescents with AIS (36 female, 12 male; mean age 11.5 ± 1.9 years; Cobb angle 10–35) were assessed for three primitive reflexes (asymmetrical tonic neck reflex [ATNR], Galant reflex, and symmetrical tonic neck reflex [STNR]) using standardized qualitative scales. Postural control was measured via computerized dynamic posturography (CDP; six sensory conditions C1–C6) and the sensory organization test (SOT). The primary analysis used hierarchical multiple regression to quantify the independent contribution of reflex retention (ATNR and Galant composite) to CDP composite score, with bootstrap resampling (5,000 iterations) to derive robust 95% confidence intervals. The STNR was analyzed separately as a specificity control. Secondary analyses examined partial correlations between individual reflexes and individual CDP conditions.

**Results:**

Primitive reflex retention was highly prevalent: 87.2% of participants ( N=47 with complete reflex data) retained at least one reflex, and 63.8% retained both. Age alone explained 25.5% of CDP composite variance ( p<0.001 ). After controlling for age and Cobb angle, reflex total score independently accounted for an additional 7.4% of variance ( $\Delta R^{2}=0.074$ , β=−6.18 , p=0.032 , 95% bootstrap CI [ − 13.77, − 0.78]). The Galant reflex exhibited the strongest independent associations with CDP composite (partial r=−0.35 , p=0.016 ) and the platform-tilt condition C4 (partial r=−0.36 , p=0.013 ). Reflex-positive participants ( ≥ 2 retained reflexes) demonstrated clinically meaningful deficits in postural control compared to reflex-negative participants (Cohen’s d=−0.63 ).

**Conclusion:**

Retained primitive reflexes, particularly the Galant reflex, are independently associated with impaired postural control in AIS beyond age and curve severity. These findings identify primitive reflex retention as a potential neurodevelopmental marker of postural dysfunction within the AIS population.

## Introduction

Adolescent idiopathic scoliosis (AIS) is a three-dimensional spinal deformity affecting 2–3% of adolescents, with onset typically occurring during the pubertal growth spurt (1–3). While the etiology is considered multifactorial, converging evidence implicates central nervous system dysfunction in both the pathogenesis and clinical presentation of AIS, including impaired proprioception, aberrant vestibular processing, and deficits in postural control (4–7).

Computerized dynamic posturography (CDP) studies have documented balance deficits in patients with AIS, particularly under sensory conditions that challenge vestibular and somatosensory integration (8–12). The sensory organization test (SOT) frequently demonstrates abnormal visual dependence and compromised vestibular function in these patients (13, 14). However, there is substantial inter-individual variability in postural control among AIS patients that is not explained by curve magnitude alone (9, 15), suggesting the presence of uncharacterized neurodevelopmental factors that modulate sensorimotor function.

Primitive reflexes are stereotyped, brainstem- and spinal cord-mediated motor responses that are present at birth and are progressively inhibited by maturing cortical and subcortical structures during the first year of life (16). Their persistence beyond early childhood, reflecting incomplete central nervous system maturation, has been associated with impaired motor coordination, postural control, and sensory integration across a range of neurodevelopmental conditions, including attention-deficit/hyperactivity disorder (ADHD), developmental coordination disorder (DCD), and dyslexia (17–21). In typically developing children, retained primitive reflexes correlate with reduced balance performance and motor proficiency (22).

Separate lines of evidence have linked AIS to structural and functional brain abnormalities, including asymmetries in brainstem nuclei, cerebellar volumes, and cortical sensorimotor processing (23–28). These neuroanatomical alterations suggest a neurodevelopmental component to AIS that may manifest functionally as persistent primitive reflex activity. However, to our knowledge, no study has examined the relationship between primitive reflex retention and postural control within the AIS population.

Investigating this relationship presents three methodological challenges. First, postural control matures substantially throughout adolescence; age is therefore a strong confound that must be rigorously controlled to isolate the specific contribution of reflex retention. Second, primitive reflex assessment is inherently qualitative and examiner-dependent, requiring careful standardization and reliability protocols. Third, the absence of population-level normative data on primitive reflex retention rates in healthy adolescents complicates the interpretation of prevalence estimates derived from clinical samples.

We hypothesized that greater primitive reflex retention would be independently associated with poorer postural control after controlling for age and curve severity.

Two considerations motivated this hypothesis. First, the Galant reflex is mediated at spinal segments T7–L1, which correspond anatomically to the thoracolumbar region most commonly affected in AIS curvature, suggesting a plausible segmental mechanism linking persistent spinal-level reflex activity to impaired postural control. Second, retained primitive reflexes in other neurodevelopmental populations have been associated with deficits specifically under challenging sensory conditions that require multisensory integration, a pattern that implicates higher-order sensorimotor dysfunction rather than a modality-specific deficit.

Accordingly, the present study aimed to: (1) characterize the prevalence of retained primitive reflexes-asymmetrical tonic neck reflex (ATNR), Galant, and symmetrical tonic neck reflex (STNR)- in a well-phenotyped AIS cohort; and (2) whether reflex retention independently predicts postural control impairment beyond age and Cobb angle, using bootstrap-validated confidence intervals; and (3) examine whether specific reflexes (particularly the Galant reflex) show condition-specific associations with individual CDP sensory conditions.

## Materials and methods

### Participants

This cross-sectional study included 48 adolescents (36 female, 12 male) with a confirmed diagnosis of AIS, recruited from the Clinical Biomechanics Assessment Laboratory, Department of Rehabilitation Medicine, Suzhou Hospital, Affiliated Hospital of Medical School, Nanjing University. A total of 62 patients were screened; 14 were excluded (12 with primary Cobb angle < 10 ° and 2 aged > 16 years [one aged 18, one aged 19]), resulting in the final analytic sample of 48. The 19-year-old patient was excluded prior to data entry and is not represented in the dataset. Inclusion criteria were: (1) diagnosis of AIS with primary Cobb angle ≥ 10° on standing posteroanterior radiograph; (2) age ≤ 16 years; and (3) no history of spinal surgery. Exclusion criteria were: (1) non-idiopathic scoliosis (congenital, neuromuscular, or syndromic etiology); (2) diagnosed neurological disorder unrelated to scoliosis; (3) lower limb length discrepancy exceeding 2 cm; and (4) acute musculoskeletal injury (defined as any fracture, ligament sprain, muscle strain, or joint injury sustained within the preceding 6 weeks that would impair the ability to walk independently or maintain prolonged standing required for the CDP testing protocol).

A participant flow diagram is provided in the Supplementary Figure S1.

The study was approved by the Medical Ethics Committee of Suzhou Hospital, Affiliated Hospital of Medical School, Nanjing University (Suzhou Science & Technology Town Hospital) (Approval No. IRB2025076). Written informed consent was obtained from all participants and their legal guardians in accordance with the Declaration of Helsinki.

### Measures

#### Demographic and radiographic characteristics

Age, sex, height, weight, and body mass index (BMI) were recorded. Standing posteroanterior full-spine radiographs were acquired using a standardized protocol. All radiographic parameters were measured by a single experienced rehabilitation physician (SW) using Surgimap software (version 2.3.2.1, Nemaris Inc., USA). The examiner was blinded to all CDP and reflex assessment results at the time of measurement. Measurements included primary Cobb angle, Risser stage (skeletal maturity, 0–5+), C7–CSVL (central sacral vertical line) offset, and apical vertebral translation (AVT).

#### Primitive reflex assessment

Three primitive reflexes were assessed by two trained examiners (Ningnan Zhu and Sheng Wang) using standardized qualitative examination procedures based on established protocols (29, 30): the ATNR, Galant reflex, and STNR. Each reflex was scored bilaterally (left and right sides independently); the two examiners reached consensus on the final score for each side, which was then averaged to yield a single reflex score:

- Asymmetrical Tonic Neck Reflex (ATNR): The participant stood with arms extended forward at shoulder height and eyes closed. The examiner passively rotated the participant’s head to one side and held the position for 5 s. A positive response was defined as involuntary abduction or flexion of the ipsilateral arm, or extension of the contralateral arm.
- Galant Reflex: The participant lay prone with arms at the sides. The examiner applied light tactile stimulation by stroking the paravertebral skin approximately 3 cm lateral to the spinous processes, from the thoracic region caudally to the lumbar region. A positive response was defined as lateral trunk flexion with hip hiking toward the stimulated side.
- Symmetrical Tonic Neck Reflex (STNR): The participant assumed a quadruped (all-fours) position. The examiner passively flexed the participant’s head for 5 s, then passively extended the head for 5 s. A positive response was defined as arm flexion coupled with leg extension during head flexion, or arm extension coupled with leg flexion during head extension.

Each reflex was rated on a five-point ordinal scale following established protocols (29, 30): 0 (absent, fully integrated), 1 (low activity), 2 (medium activity), 3 (high activity), 4 (maximum activity). The final score for each reflex was the mean of the left-side and right-side consensus scores.

The STNR was assessed as part of the standardized reflex battery. Because the STNR is mediated at upper cervical segments (C5–C6) whereas the Galant reflex (the primary reflex of mechanistic interest) is mediated at thoracolumbar segments [T7–L1 (16)], the STNR was analyzed separately to preserve segmental specificity of the primary hypothesis. The STNR was excluded from the primary composite score; complete STNR results are reported in the Supplementary Table S3. A composite reflex total score was calculated as the mean of the two hypothesis-relevant reflex scores (ATNR and Galant), yielding a continuous score ranging from 0 to 4 (30). For secondary categorical analyses, participants were classified as “reflex-positive” if they retained both ATNR and Galant reflexes with a score ≥ 1 and “reflex-negative” otherwise.

#### Postural control assessment

Postural control was assessed using computerized dynamic posturography (Smart Balance Master, NeuroCom International, Clackamas, OR, USA). The standard NeuroCom Sensory Organization Test (SOT) protocol was administered in accordance with the manufacturer’s guidelines. Prior to formal data collection, each participant completed one familiarization trial per sensory condition (C1–C6) to minimize learning effects; these trials were not included in the analysis. Each condition was then administered in triplicate (three 20-s trials): C1 (eyes open, fixed support surface and visual surround), C2 (eyes closed, fixed support surface), C3 (eyes open, fixed support surface, sway-referenced visual surround), C4 (eyes open, sway-referenced support surface, fixed visual surround), C5 (eyes closed, sway-referenced support surface), and C6 (eyes open, sway-referenced support surface and visual surround).

The equilibrium score (EQ) for each condition was computed from the maximum peak-to-peak anteroposterior sway angle relative to the theoretical limits of stability, ranging from 0 (loss of balance/fall) to 100 (perfect stability) (31); see Figure 1 for SOT conditions. A weighted CDP composite equilibrium score was derived across all six conditions.

**Figure 1:**
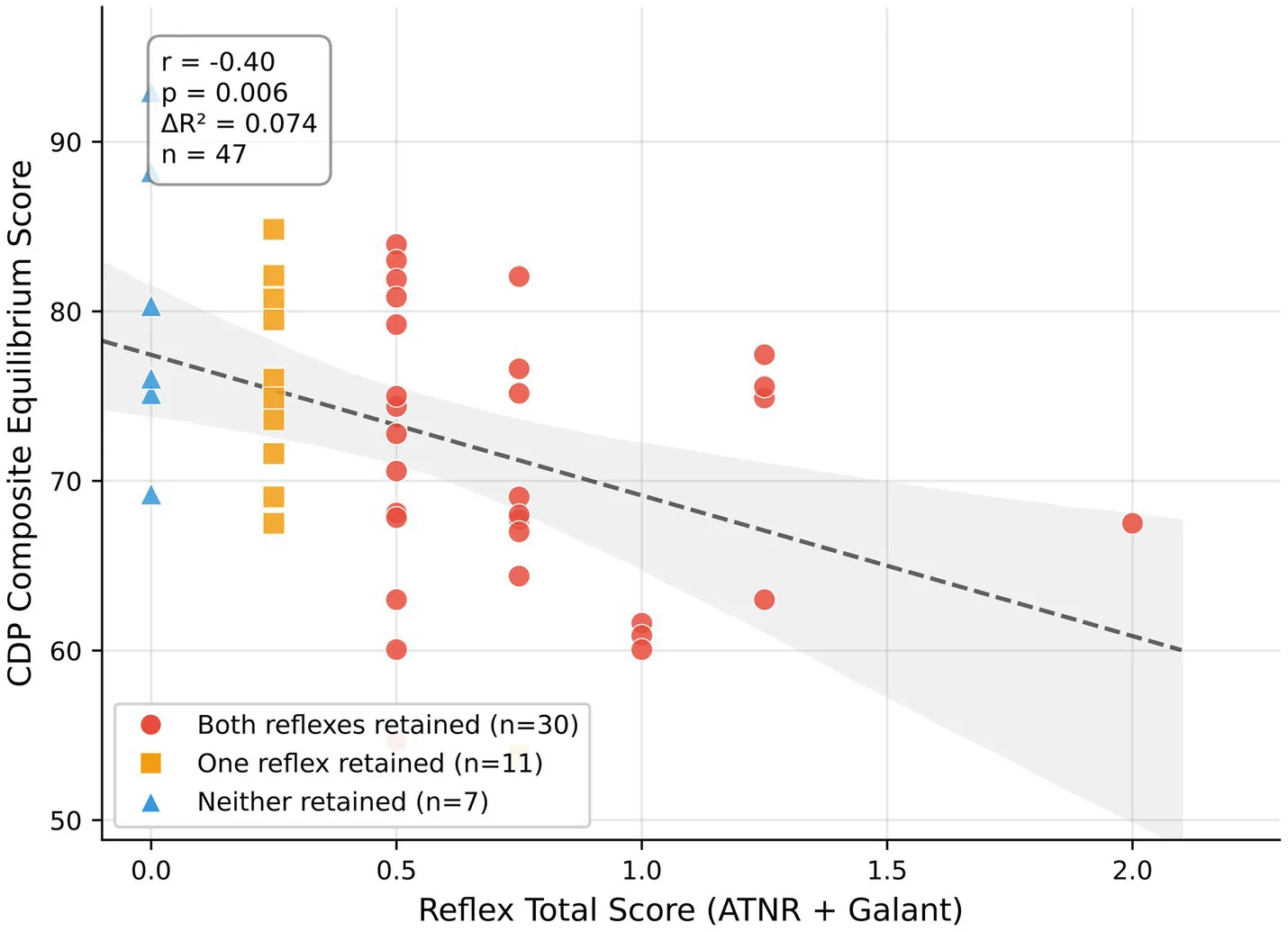
Higher primitive reflex retention is associated with poorer postural control in adolescents with idiopathic scoliosis. Scatter plot shows CDP composite score versus reflex total score ( n=47 ). The solid line represents the ordinary least squares regression fit; the shaded band represents the 95% bootstrap confidence interval (5,000 resamples). Each point represents one participant.

SOT sensory analysis ratios were calculated to estimate the functional contribution of each sensory system to postural control (31): somatosensory ratio (SOM: C2/C1), visual ratio (VIS: C4/C1), vestibular ratio (VEST: C5/C1), and visual preference ratio (PREF: [C3 + C6] / [C2 + C5]). SOT ratios below 85 were considered clinically abnormal based on the manufacturer’s normative database.

#### Vestibular screening

Vestibular function was screened using the Fukuda stepping test (angle of unintentional rotation after 50 steps marching in place with eyes closed and arms extended). Motion sickness history was obtained via self-report questionnaire.

### Statistical analysis

Descriptive statistics are reported as mean ± standard deviation for continuous variables and frequencies (percentages) for categorical variables. Distributional properties were examined using histograms, Q–Q plots, and Shapiro–Wilk tests. Variables with non-normal distributions were analyzed using both parametric and non-parametric methods; results were concordant in all cases.

#### Primary analysis

The primary hypothesis was tested using hierarchical multiple linear regression with CDP composite score as the dependent variable. Predictors were entered in three sequential blocks: (1) age, (2) Cobb angle, and (3) reflex total score. Age was entered first because it is the strongest known predictor of postural control in adolescents, accounting for developmental maturation. Cobb angle was entered second to address the alternative explanation that reflex retention merely proxies for curve severity. Reflex total score was entered last to provide the most conservative test of its independent contribution. The incremental variance explained (ΔR^2^) at each step was evaluated using F-tests for change in R^2^. Regression assumptions (linearity, homoscedasticity, normality of residuals, absence of multicollinearity) were verified using residual-versus-fitted plots, Q–Q plots of residuals, and variance inflation factors (VIF < 2 for all predictors). BMI, Risser stage, and sex were considered as potential covariates but excluded: BMI because its association with postural control in adolescents is weak and inconsistent; Risser stage because of high collinearity with age (r > 0.80); and sex because regression residuals showed no systematic sex effects.

To assess the robustness of the primary finding against distributional assumptions, bootstrap resampling (5,000 iterations with replacement) was performed to derive bias-corrected 95% confidence intervals for the regression coefficient of reflex total score on CDP composite, controlling for age and Cobb angle.

#### Secondary analyses

Partial correlations (Pearson r , controlling for age and Cobb angle via residualization) were computed between individual reflex scores and individual CDP condition scores, with bootstrap 95% confidence intervals (2,000 iterations per pair). Associations whose bootstrap CIs excluded zero were considered robust.

An exploratory between-group comparison (reflex-positive vs. reflex-negative) was performed using Welch’s independent-samples t -test, with effect size quantified by Cohen’s d . Group differences adjusted for age and Cobb angle were examined via analysis of covariance (ANCOVA).

To contextualize the sensorimotor specificity of the reflex–balance association, the partial correlation between reflex total score and SOT vestibular ratio (controlling for age and Cobb angle) was examined.

### Multiple comparison correction

Given the exploratory nature of the secondary partial correlation analyses (28 tests across reflex–CDP condition pairs), both uncorrected p -values and Benjamini–Hochberg false discovery rate (FDR)-adjusted q -values are reported. However, the primary analysis (single hierarchical regression with three planned steps) did not require multiplicity correction.

All analyses were performed in Python 3.11 using pandas, NumPy, SciPy, and statsmodels. Statistical significance was set at α=0.05 (two-tailed).

## Results

### Sample characteristics

The final analytic sample comprised 48 participants (36 female, 12 male) after application of inclusion/exclusion criteria (Table 1). Mean age was 11.5 ± 1.9 years (range 8–16). Mean primary Cobb angle was 16.2 ± 6.0 (range 10.0–34.7), corresponding to mild-to-moderate curve severity. Mean Risser stage was 3.8 ± 2.7, consistent with early-to-mid skeletal maturity.

**Table 1:** Participant characteristics ( N=48 ).

Characteristic	Value
Age (years), mean ± SD (range)	11.5 ± 1.9 (8.0–16.0)
Sex, female/male, n (%)	36 (75%) / 12 (25%)
BMI (kg/m^2^), mean ± SD (range)	16.9 ± 3.1 (11.4–23.8)
Primary Cobb angle (), mean ± SD (range)	16.2 ± 6.0 (10.0–34.7)
Risser stage, mean ± SD (range)	3.8 ± 2.7 (0–8)
C7–CSVL offset (mm), mean ± SD (range)	13.9 ± 8.4 (0.0–33.0)
Apical vertebral translation (mm), mean ± SD (range)	17.2 ± 8.5 (0.0–34.7)

BMI, body mass index; C7–CSVL, seventh cervical vertebra to central sacral vertical line.

### Prevalence of primitive reflex retention

Retained primitive reflexes were highly prevalent in this AIS sample (Table 2). The ATNR was retained (score ≥ 1) in 78.7% of participants and the Galant reflex in 72.3%. Overall, 87.2% of participants exhibited at least one retained reflex, and 63.8% (30/47) retained both reflexes (classified as reflex-positive). The mean reflex total score was 0.56 ± 0.41 (range 0.00–2.00).

**Table 2:** Primitive reflex retention in AIS ( N=47 with complete reflex data).

Reflex	Score (mean ± SD)	Any positive, n (%)	Score ≥ 1.0, n (%)
ATNR	0.46 ± 0.34	37 (78.7%)	4 (8.5%)
Galant	0.66 ± 0.63	34 (72.3%)	15 (31.9%)
Reflex total score	0.56 ± 0.41	41 (87.2%)	–

ATNR, asymmetrical tonic neck reflex. Any positive = score ≥ 1 on at least one side. Reflex total score = mean of ATNR and Galant reflex scores.

### Postural control profile

CDP equilibrium scores demonstrated the expected progressive decline from the least challenging (C1: 89.5 ± 5.9) to the most challenging sensory condition (C6: 50.5 ± 16.4; Table 3). The CDP composite score averaged 72.9 ± 8.5. SOT sensory analysis showed a selective pattern of impairment in sensory integration: vestibular ratios were abnormal ( < 85) in 93.8% of participants, visual ratios in 50.0%, and somatosensory ratios in none (0.0%).

**Table 3:** CDP and SOT results ( N=48 ).

Measure	Mean ± SD	Abnormal, *n* (%)
C1 (eyes open, fixed)	89.5 ± 5.9	–
C2 (eyes closed, fixed)	87.5 ± 5.4	–
C3 (sway-referenced vision)	82.2 ± 8.3	–
C4 (sway-referenced support)	71.5 ± 13.9	–
C5 (eyes closed, sway support)	55.3 ± 16.0	–
C6 (sway vision + support)	50.5 ± 16.4	–
CDP composite	72.9 ± 8.5	–
SOT somatosensory (SOM)	97.9 ± 3.8	0 (0.0%)
SOT visual (VIS)	80.0 ± 14.8	24 (50.0%)
SOT vestibular (VEST)	61.0 ± 16.8	45 (93.8%)
SOT visual preference (PREF)	92.4 ± 11.9	11 (22.9%)

CDP, computerized dynamic posturography; SOT, sensory organization test. Abnormal defined as SOT ratio < 85 (clinical cutoff derived from manufacturer normative data).

### Primary analysis: reflex retention and postural control

The zero-order Pearson correlation between reflex total score and CDP composite was r=−0.396 ( p=0.006 ), indicating a moderate negative association (Figure 1). The Spearman rank correlation ( ρ=−0.446 , p=0.002 ) confirmed robustness to non-normality.

Hierarchical regression (Table 4) showed that age entered at Step 1 accounted for 25.5% of CDP composite variance ( p<0.001 ). The addition of Cobb angle at Step 2 contributed a non-significant 2.4% incremental variance ( $\Delta R^{2}=0.024$ , p=0.235 ). Critically, reflex total score entered at Step 3 independently accounted for an additional 7.4% of variance ( $\Delta R^{2}=0.074$ , β=−6.18 , p=0.032 ), beyond the effects of age and Cobb angle. The full model was significant ( F[3,43]=7.82 , p<0.001 , adjusted $R^{2}=0.308$ ).

**Table 4:** Hierarchical multiple regression predicting CDP composite score.

Step and predictor	*β*	SE	*t*	*p*	$\Delta R^{2}$
Step 1					0.255
Age	2.27	0.58	3.93	< 0.001	
Step 2					0.024
Age	1.86	0.67	2.78	0.008	
Cobb angle	0.25	0.21	1.21	0.235	
Step 3					**0.074**
Age	1.27	0.69	1.83	0.074	
Cobb angle	0.34	0.21	1.66	0.104	
**Reflex total score**	− **6.18**	**2.79**	− **2.21**	**0.032**	

SE, standard error. Bold indicates the primary hypothesis test. Full model: F(3,43)=7.82 , p<0.001 , $R^{2}=0.353$ , adjusted $R^{2}=0.308$ .

Bootstrap validation (5,000 resamples) confirmed the robustness of the reflex–CDP association: the bias-corrected bootstrap coefficient was β=−6.41 , with a 95% confidence interval of [ − 13.77, − 0.78] that excludes zero. Thus, each one-unit increase in reflex total score (approximately the difference between having no retained reflexes and having all three) was associated with an estimated 6.2-point decrement in CDP composite score.

### Secondary analyses

#### Individual reflexes and CDP conditions

Partial correlations (controlling for age and Cobb angle) between individual reflexes and individual CDP conditions are presented in Table 5 and Figure 2. The Galant reflex showed the strongest and most consistent pattern of associations, with significant negative partial correlations with CDP composite (partial r=−0.350 , 95% bootstrap CI [ − 0.596, − 0.053], p=0.016 ) and the platform-tilt condition C4 (partial r=−0.360 , 95% CI [ − 0.625, − 0.055], p=0.013 ). The reflex total score was similarly associated with C4 (partial r=−0.340 , CI [ − 0.594, − 0.080], p=0.020 ) and CDP composite (partial r=−0.320 , CI [ − 0.578, − 0.047], p=0.028 ). Trend-level associations ( p<0.10 ) were observed between the Galant reflex and both C5 (sway-referenced support, eyes closed) and C6 (sway-referenced vision and support), suggesting that the reflex–balance association was most pronounced under the most challenging sensory conditions.

**Table 5:** Partial correlations between reflex scores and CDP conditions, controlling for age and Cobb angle.

Reflex	CDP condition	Partial *r*	95% bootstrap CI	*p*
**Galant**	**C4 (platform tilt)**	− **0.360**	**[** − **0.625,** − **0.055]**	**0.013**
**Galant**	**CDP composite**	− **0.350**	**[** − **0.596,** − **0.053]**	**0.016**
**Reflex total**	**C4 (platform tilt)**	− **0.340**	**[** − **0.594,** − **0.080]**	**0.020**
**Reflex total**	**CDP composite**	− **0.320**	**[** − **0.578,** − **0.047]**	**0.028**
Reflex total	C6 (sway vision + support)	− 0.196	[ − 0.478, + 0.105]	0.191
Galant	C5 (eyes closed + sway)	− 0.252	[ − 0.511, + 0.029]	0.087
Reflex total	C5 (eyes closed + sway)	− 0.243	[ − 0.502, + 0.019]	0.100

Only associations with p<0.10 are shown. Bold rows indicate associations with bootstrap 95% CI excluding zero. All partial correlations control for age and Cobb angle via residualization.

**Figure 2:**
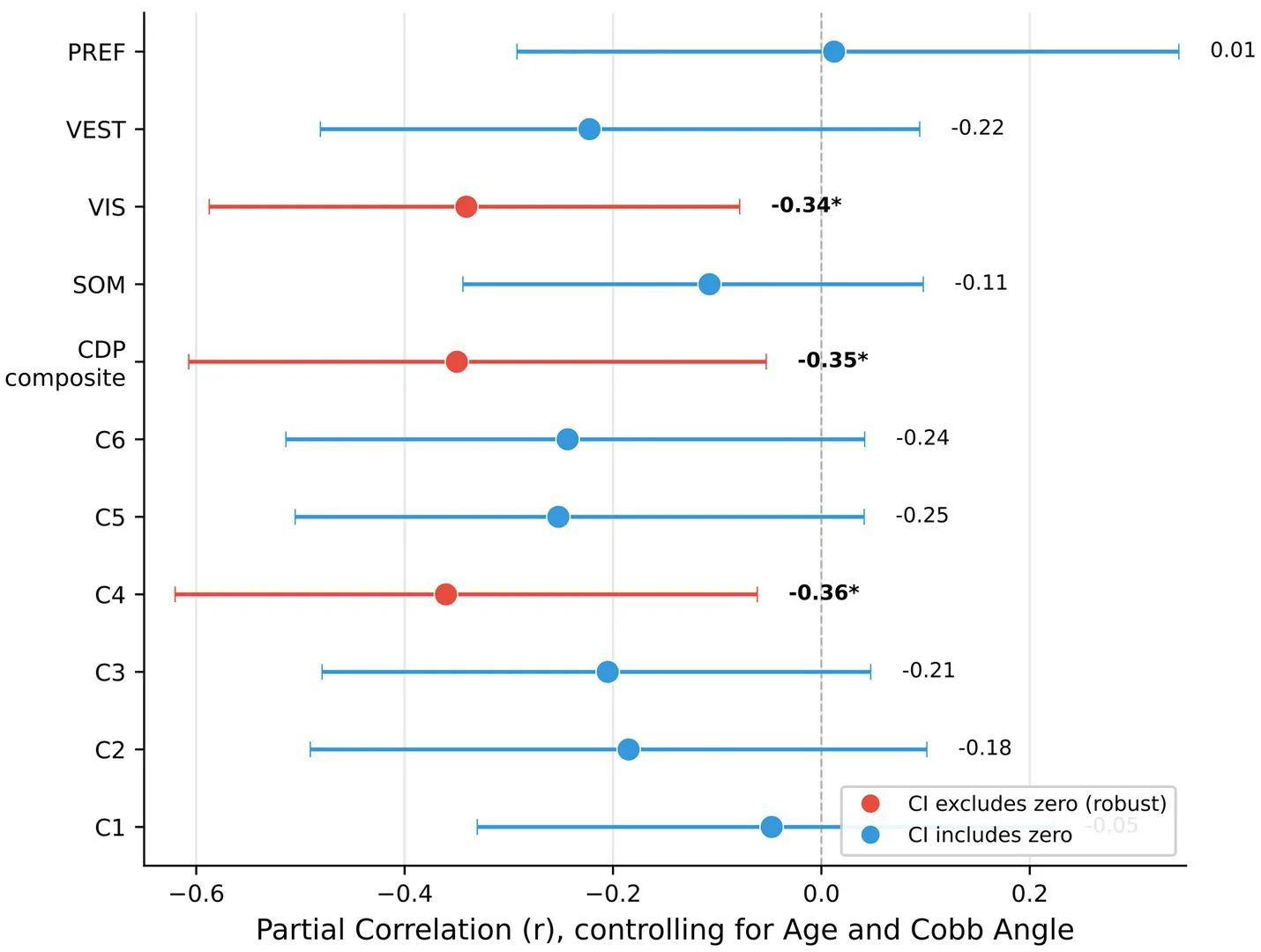
The Galant reflex shows condition-specific associations with postural control, with the strongest effect under the proprioceptively challenging C4 condition. Forest plot showing partial correlation coefficients (Pearson r ) between Galant reflex score and equilibrium scores for each CDP condition, controlling for age and Cobb angle. Error bars represent 95% bootstrap confidence intervals (2,000 resamples). Asterisks (*) denote associations whose bootstrap confidence interval excludes zero. C1: yes open, fixed support; C2: yes closed, fixed support; C3: way-referenced vision, fixed support; C4: way-referenced support, fixed vision; C5: yes closed, sway-referenced support; C6: way-referenced vision and support.

The ATNR exhibited a consistently negative but weaker pattern (all ∣pr∣<0.14 , all p>0.35 ).

After Benjamini–Hochberg FDR correction across all 21 reflex–CDP partial correlation tests, none of the associations survived correction (all FDR q>0.14 ). These conservative FDR results should be interpreted in light of the correlated nature of CDP conditions and the limited sample size; bootstrap confidence intervals serve as the primary robustness indicator.

#### Reflex-positive vs. reflex-negative dichotomous comparison

Reflex-positive participants ( ≥ 2 retained reflexes, n=30 ) had significantly lower CDP composite scores than reflex-negative participants ( < 2 retained reflexes, n=17 ; 70.9 ± 7.9 vs. 76.2 ± 9.0; Welch’s t[32.5]=−2.04 , p=0.050 ). This difference corresponded to a medium-to-large effect size (Cohen’s d=−0.63 , 95% CI [ − 1.24, − 0.02]). After adjusting for age and Cobb angle via ANCOVA, the adjusted mean difference was attenuated (adjusted β=−3.95 , p=0.099 ), consistent with partial confounding by age-related maturation.

#### Reflexes and SOT sensory ratios

The reflex total score showed a weak negative partial correlation with the SOT vestibular ratio that did not reach statistical significance (partial r=−0.198 , 95% bootstrap CI [ − 0.473, + 0.091], p=0.181 ). This suggests that the deleterious association between reflex retention and postural control is not exclusively mediated by vestibular dysfunction as captured by the SOT vestibular ratio, and may reflect broader deficits in multisensory integration.

## Discussion

This study provides the first evidence that retained primitive reflexes are independently associated with impaired postural control in adolescents with idiopathic scoliosis. The primary finding, that reflex retention explains approximately 7.4% of CDP composite variance beyond age and curve severity, was robust to bootstrap resampling and consistent across multiple analytical approaches. These results identify primitive reflex retention as a potential neurodevelopmental marker of postural dysfunction in AIS.

### Primitive reflex retention as a neurodevelopmental marker in AIS

The high prevalence of retained primitive reflexes observed in this sample (87.2% with at least one retained reflex, 63.8% retaining both ATNR and Galant) warrants cautious interpretation. The absence of a matched healthy control group precludes any conclusion about whether these rates are higher than in the general adolescent population; normative data on primitive reflex retention in adolescents are scarce (32). Nevertheless, the internal association between reflex retention and postural control within the AIS cohort itself carries implications independent of normative comparison, as we elaborate below.

The hierarchical regression results illuminate the relative contributions to postural control in AIS. Age was the dominant predictor ( $R^{2}=0.255$ ), consistent with the well-established maturation of postural control systems throughout adolescence (33). Cobb angle (the primary clinical metric of curve severity) contributed minimally and non-significantly ( $\Delta R^{2}=0.024$ ), replicating previous findings that static radiographic curve magnitude is a poor predictor of functional balance in mild-to-moderate AIS (9, 15). In contrast, reflex retention independently accounted for more than three times the variance explained by Cobb angle ( $\Delta R^{2}=0.074$ ), suggesting that neurodevelopmental factors captured by primitive reflex status may be more informative for postural function than the radiographic severity of the spinal curvature itself.

### Specificity of the Galant reflex and challenging sensory conditions

Several aspects of the observed pattern suggest mechanistic specificity beyond a general association between neurodevelopmental immaturity and motor function.

First, the Galant reflex emerged as the strongest and most consistent predictor among the reflexes examined. The Galant reflex is mediated at spinal segments T7–L1 (16), the same thoracolumbar region most commonly involved in AIS curvature. Its persistence indicates incomplete descending inhibition of spinal reflex circuits by brainstem and cortical pathways (34). The segmental correspondence between the Galant reflex circuit and the anatomical site of spinal deformity raises the hypothesis that persistent segmental reflex activity may contribute to asymmetric paraspinal muscle activation patterns that influence curve development or progression. This hypothesis cannot be tested in the current cross-sectional design but merits longitudinal investigation.

Second, the association was most pronounced under the most challenging CDP sensory conditions (C4–C6), which require multisensory integration under sensory conflict. The Galant reflex showed significant partial correlations with C4 (platform tilt, pr=−0.36 ) and trend-level associations with C5 and C6. This gradient, weakest in simple conditions and strongest in complex conditions, is consistent with the hypothesis that retained primitive reflexes reflect impaired higher-order sensorimotor integration rather than a modality-specific deficit. The absence of a significant association between reflex scores and the SOT vestibular ratio ( pr=−0.19 , n.s.) further supports this interpretation: the reflex–balance association is not simply a reflection of vestibular dysfunction.

Third, the effect of reflex retention was independent of age and Cobb angle, demonstrating that reflex status is not merely a proxy for overall neurodevelopmental immaturity (which would be captured by age) or scoliosis severity (which would be captured by Cobb angle). This independence supports the notion that retained primitive reflexes may delineate a distinct neurodevelopmental phenotype within the heterogeneous AIS population.

Fourth, the near-complete integration of the STNR (included in reflex assessment but excluded from the primary composite score; see Supplementary material) provides an internal specificity control. If the association between retained reflexes and postural control reflected a generalized neurodevelopmental delay, all three reflexes would show comparable retention rates and patterns of association. Instead, the STNR was selectively absent: its mean score was 0.05 ± 0.16 (median = 0.00), with 95.7% of all tested sides scoring zero and only 8.3% (4/48) of participants showing any STNR response ( ≥ 1 on either side). This pattern was strikingly different from the ATNR (77.1% retained) and Galant reflex (70.8% retained). The STNR showed negligible zero-order correlation with CDP composite ( r=−0.02 , p=0.91 ) and contributed near-zero incremental variance ( $\Delta R^{2}=0.003$ , p=0.69 ), and its scores were uncorrelated with both ATNR ( ρ=−0.22 , n.s.) and Galant reflex ( ρ=−0.14 , n.s.). The anatomy of these reflexes further supports the specificity argument: the STNR is mediated at upper cervical segments (C5–C6), the ATNR engages brainstem–cervical circuits, and the Galant reflex is mediated at thoracolumbar segments [T7–L1 (16)] that correspond anatomically to the region most commonly affected by scoliotic curvature. The selective retention of reflexes with thoracolumbar or whole-body involvement, along with the complete integration of the purely cervical STNR, is consistent with the hypothesis that AIS-associated neurodevelopmental alterations disproportionately affect spinal and brainstem circuits governing trunk postural control, rather than reflecting a global maturational delay.

### Convergence with existing literature

While this is, to our knowledge, the first study to link primitive reflex retention to postural control in AIS, a single prior study has examined primitive reflex retention in an AIS cohort. Vlădăreanu et al. (35) assessed ATNR, STNR, Galant (termed GSR), and Moro reflexes in 162 Romanian patients aged 7–19 years with idiopathic scoliosis. Their study reported that Galant reflex retention was significantly correlated with Cobb angle ( r=0.233 , p=0.011 ), and that all reflex scores decreased over 12 months of conservative treatment. However, the reflex retention rates reported by Vlădăreanu et al. (35) differ markedly from those observed in the present study: their ATNR retention was 38.3% (vs. 77.1% here), Galant 27.8% (vs. 70.8%), and STNR 44.4% (vs. 8.3%). These discrepancies almost certainly reflect differences in reflex assessment methodology; Vlădăreanu et al. (35) did not specify a validated 0–4 grading protocol comparable to the Pecuch scale employed here (30), rather than genuine population-level differences. The consistent finding across both studies is that Galant reflex retention is the reflex most strongly associated with scoliosis-related outcomes, whether structural (Cobb angle) or functional (postural control). Importantly, the study by Vlădăreanu et al. (35) did not assess postural control or balance, so the present findings are not redundant with their work.

More broadly, the present findings are congruent with two established lines of evidence. On one hand, retained primitive reflexes have been consistently associated with impaired balance, coordination, and sensory integration in children with neurodevelopmental conditions including DCD, ADHD, and dyslexia (17, 19, 20, 22). A recent systematic review by Provazník et al. (21) confirmed that persisting primitive reflexes are reliably associated with motor and cognitive developmental outcomes across heterogeneous pediatric populations. On the other hand, AIS has been linked to structural and functional brain abnormalities, including asymmetries in vestibular nuclei, brainstem, and cerebellum (23, 25, 26, 28), as well as altered cortical sensorimotor representations and white matter connectivity (24, 27) that imply a neurodevelopmental contribution to the condition. Our findings bridge these two literatures by demonstrating that a clinically accessible marker of brainstem-level dysfunction (primitive reflex retention) correlates with a functionally relevant outcome (postural control) in AIS.

Regarding normative context, the primitive reflex retention rates observed in the present AIS sample are substantially higher than those reported in healthy preschool and school-age children using comparable assessment methods. Pecuch et al. (30) found that in healthy children aged 4–6 years, primitive reflex activity (graded on the same 0–4 scale) declined progressively with age and was markedly lower than the levels observed in our adolescent AIS cohort, whose mean age was 11.5 years, an age at which near-complete reflex integration would be expected in typically developing children (22). However, without a matched healthy control group assessed in parallel using identical methodology, this comparison remains provisional.

### Clinical implications

If replicated in larger, independent samples, these findings carry several clinical implications:

Screening and phenotyping: Primitive reflex assessment requires no specialized equipment, can be completed in under five minutes, and can be performed by trained clinicians across disciplines (physicians, physical therapists, occupational therapists). Incorporating reflex screening into existing AIS evaluation protocols could enable identification of a neurodevelopmentally distinct subgroup that may benefit from targeted intervention.

Stratification in research: The substantial heterogeneity in postural control outcomes among AIS patients (36) has complicated the interpretation of balance rehabilitation trials. Reflex status may serve as a biologically meaningful stratification variable that reduces unexplained variance and enhances statistical power to detect treatment effects in reflex-positive subgroups.

Therapeutic implications: Reflex integration programs, involving repetitive, patterned movements designed to facilitate inhibition of retained primitive reflexes, have demonstrated benefits for motor coordination and academic performance in children with DCD and ADHD (19, 37, 38). Whether such interventions can improve postural control in reflex-positive AIS patients, either as standalone therapy or as an adjunct to physiotherapeutic scoliosis-specific exercises (PSSE), is an open question that warrants controlled investigation.

### Limitations

Several limitations of this study should be carefully considered.

First, the absence of a healthy control group is the primary limitation. The internal association between reflex retention and postural control within AIS is the central finding and is valid regardless of normative prevalence. However, the absence of control data means we cannot determine whether the observed retention rate of 87.2% differs from that in the general adolescent population. Future studies should include age- and sex-matched healthy comparators to establish whether primitive reflex retention is disproportionately prevalent in AIS or simply co-occurs with postural deficits at similar rates across populations.

Second, the sample size ( N=48 ), while comparable to or exceeding many CDP studies in AIS, limits statistical power for detecting smaller effects and precludes more sophisticated multivariate approaches (e.g., testing interaction effects, mediation models, or machine learning-based phenotyping). Bootstrap confidence intervals provide reassurance regarding the robustness of the primary finding, but larger multi-center samples are needed to fully characterize the reflex–balance relationship.

Third, the cross-sectional design cannot establish temporality or causality. It is unknown whether retained reflexes contribute to postural control deficits (and potentially to scoliosis progression), whether both are downstream manifestations of a common neurodevelopmental pathway, or whether the association is bidirectional. Longitudinal studies tracking reflex integration and postural control from early childhood through adolescence are required to disentangle these possibilities.

Fourth, reflex assessment was qualitative and examiner-dependent. Although two trained examiners independently scored each reflex and used a standardized protocol, inter-rater reliability was not formally quantified. Objective quantification using surface electromyography, inertial measurement units, or motion capture would enhance measurement precision and reproducibility in future work.

Fifth, the SOT vestibular ratio exhibited near-ceiling impairment (93.8% abnormal), limiting its utility as a continuous predictor variable. More sensitive vestibular assessments, such as video head impulse testing (vHIT), vestibular evoked myogenic potentials (VEMPs), or subjective visual vertical, would better characterize the specific vestibular contributions to the reflex–balance relationship.

### Future directions

Beyond addressing the limitations outlined above, several lines of investigation are suggested by these preliminary findings. First, a normative study should establish the prevalence of retained primitive reflexes across a large, demographically representative sample of healthy adolescents to provide the population benchmarks currently lacking. Second, longitudinal cohorts tracking reflex integration, postural control, and curve progression from pre-pubertal screening through skeletal maturity are needed to disentangle the temporal relationships among these variables. Third, neuroimaging studies should examine whether reflex-positive AIS patients exhibit distinct patterns of brainstem, cerebellar, or cortical structure and function. Fourth, randomized controlled trials should test whether reflex integration therapy improves postural control in reflex-positive AIS patients. Finally, multi-site replication in geographically and ethnically diverse samples is essential to confirm the generalizability of these preliminary findings.

## Conclusion

In this cross-sectional study of 48 adolescents with idiopathic scoliosis, retained primitive reflexes, in particular the Galant reflex, were independently associated with impaired postural control, beyond the effects of age and curve magnitude. The reflex–balance relationship was most pronounced under challenging sensory conditions requiring multisensory integration, and was not attributable to isolated vestibular dysfunction. These findings provide preliminary evidence that primitive reflex retention may represent a clinically accessible, neurodevelopmentally meaningful marker of postural dysfunction in AIS. Primitive reflex screening warrants consideration as a complementary assessment tool for neurodevelopmental phenotyping in this population, with potential implications for risk stratification and targeted intervention.

## Data Availability

The original contributions presented in the study are included in the article/Supplementary material, further inquiries can be directed to the corresponding authors.
