# Supplementary material for: Retained primitive reflexes are independently associated with impaired postural control in adolescents with idiopathic scoliosis
Source: Front Neurol. 2026 Sep 14;17:1921169. doi: 10.3389/fneur.2026.1921169 (PMC13616718; doi:10.3389/fneur.2026.1921169)
Supplement: Supplementary file 1 [file Table_1.DOCX]

Patient-level data: CDP equilibrium scores and SOT ratios (Panel B).

| **ID** | **C1** | **C2** | **C3** | **C4** | **C5** | **C6** | **CDP** | **SOM** | **VIS** | **VEST** | **PREF** |
| --- | --- | --- | --- | --- | --- | --- | --- | --- | --- | --- | --- |
|  |  |  |  |  |  |  | **comp** |  |  |  |  |
| 1 | 93.3 | 84.7 | 87.0 | 82.0 | 52.7 | 52.3 | 75.3 | 91 | 88 | 57 | 101 |
| 2 | 89.7 | 88.7 | 87.3 | 79.0 | 36.0 | 33.7 | 69.1 | 99 | 88 | 40 | 97 |
| 3 | 92.3 | 86.7 | 79.0 | 62.7 | 54.3 | 71.3 | 74.4 | 95 | 68 | 59 | 106 |
| 4 | 87.7 | 86.3 | 85.0 | 75.0 | 45.5 | 71.3 | 75.1 | 98 | 85 | 34 | 134 |
| 5 | 87.0 | 84.0 | 65.3 | 59.7 | 43.0 | 30.7 | 61.6 | 97 | 68 | 49 | 76 |
| 6 | 95.0 | 94.3 | 88.7 | 58.7 | 43.0 | 35.7 | 69.2 | 99 | 62 | 45 | 91 |
| 7 | 91.3 | 88.7 | 83.7 | 67.3 | 71.0 | 73.3 | 79.2 | 98 | 74 | 78 | 98 |
| 8 | 89.3 | 87.7 | 89.3 | 60.0 | 31.0 | 29.0 | 64.4 | 99 | 67 | 35 | 99 |
| 9 | 91.7 | 88.7 | 81.0 | 68.3 | 63.3 | 48.7 | 73.6 | 97 | 74 | 68 | 86 |
| 10 | 93.0 | 90.3 | 93.3 | 79.7 | 82.0 | 65.3 | 83.9 | 97 | 86 | 88 | 92 |
| 11 | 80.3 | 76.7 | 76.0 | 71.7 | 53.3 | 50.7 | 68.1 | 96 | 90 | 66 | 98 |
| 12 | 86.0 | 88.3 | 82.7 | 66.0 | 55.7 | 26.3 | 67.5 | 102 | 77 | 65 | 76 |
| 13 | 83.7 | 86.0 | 65.3 | 63.7 | 64.7 | 43.7 | 67.8 | 102 | 76 | 73 | 74 |
| 14 | 93.0 | 88.3 | 85.3 | 84.0 | 46.3 | 54.0 | 75.2 | 95 | 90 | 49 | 104 |
| 15 | 94.7 | 96.7 | 90.3 | 88.7 | 74.3 | 53.3 | 83.0 | 102 | 94 | 78 | 84 |
| 16 | 90.3 | 89.0 | 86.0 | 53.3 | 36.3 | 23.0 | 63.0 | 99 | 59 | 40 | 87 |
| 17 | 88.7 | 89.0 | 73.3 | 47.3 | 67.3 | 48.7 | 69.1 | 100 | 53 | 75 | 78 |
| 18 | 78.7 | 79.7 | 69.3 | 59.0 | 35.7 | 43.0 | 60.9 | 101 | 75 | 46 | 97 |
| 19 | 95.0 | 90.0 | 87.3 | 78.3 | 68.7 | 72.0 | 81.9 | 95 | 82 | 73 | 100 |
| 20 | 93.7 | 89.0 | 85.0 | 72.0 | 72.7 | 47.3 | 76.6 | 95 | 77 | 78 | 81 |
| 21 | 93.3 | 92.7 | 84.7 | 86.0 | 74.0 | 61.7 | 82.1 | 100 | 92 | 80 | 88 |
| 22 | 95.3 | 92.3 | 90.3 | 82.7 | 77.3 | 71.0 | 84.8 | 97 | 87 | 81 | 95 |
| 23 | 94.7 | 92.0 | 94.0 | 84.7 | 56.0 | 63.7 | 80.8 | 97 | 89 | 59 | 107 |
| 24 | 89.0 | 86.0 | 80.3 | 55.0 | 44.3 | 50.3 | 67.5 | 97 | 62 | 49 | 100 |
| 25 | 88.0 | 86.7 | 81.0 | 67.0 | 63.7 | 63.7 | 75.0 | 99 | 76 | 73 | 96 |
| 26 | 92.0 | 94.0 | 90.7 | 85.3 | 81.7 | 38.3 | 80.3 | 102 | 92 | 89 | 73 |
| 27 | 74.0 | 70.0 | 65.0 | 72.7 | 50.0 | 46.3 | 63.0 | 95 | 99 | 68 | 92 |
| 28 | 91.7 | 85.3 | 68.3 | 42.7 | 35.7 | 36.7 | 60.1 | 92 | 47 | 39 | 87 |
| 29 | 94.3 | 90.3 | 87.0 | 81.7 | 57.3 | 54.0 | 77.4 | 96 | 87 | 61 | 96 |
| 30 | 91.3 | 89.0 | 85.0 | 82.7 | 44.3 | 37.3 | 71.6 | 98 | 91 | 48 | 92 |
| 31 | 88.0 | 90.0 | 84.3 | 69.7 | 65.0 | 52.3 | 74.9 | 102 | 80 | 74 | 88 |
| 32 | 93.7 | 93.3 | 93.3 | 78.0 | 45.3 | — | 80.7 | 99 | 83 | 48 | 67 |
| 33 | 82.7 | 84.3 | 85.7 | 58.0 | 20.3 | 29.3 | 60.1 | 101 | 70 | 24 | 111 |
| 34 | 90.3 | 82.7 | 61.7 | 25.3 | 32.7 | 30.3 | 53.8 | 92 | 28 | 37 | 79 |
| 35 | 93.3 | 91.3 | 83.3 | 79.0 | 70.0 | 60.0 | 79.5 | 98 | 85 | 75 | 89 |
| 36 | 88.3 | 82.3 | 78.0 | 64.3 | 47.3 | 46.0 | 67.7 | 93 | 73 | 53 | 96 |
| 37 | 89.7 | 82.7 | 74.7 | 60.0 | 52.3 | 48.7 | 68.0 | 92 | 67 | 58 | 92 |
| 38 | 83.7 | 86.3 | 86.7 | 81.3 | 66.0 | 52.0 | 76.0 | 102 | 96 | 79 | 91 |
| 39 | 65.7 | 74.0 | 68.3 | 64.7 | 27.7 | 27.7 | 54.7 | 112 | 98 | 42 | 94 |
| 40 | 90.3 | 83.7 | 80.7 | 85.3 | 44.7 | 52.0 | 72.8 | 93 | 94 | 50 | 90 |
| 41 | 89.0 | 83.7 | 84.3 | 58.7 | 59.3 | 27.0 | 67.0 | 94 | 66 | 66 | 78 |
| 42 | 91.3 | 89.7 | 77.3 | 75.0 | 49.0 | 67.0 | 74.9 | 99 | 82 | 54 | 104 |
| 43 | 90.0 | 93.7 | 90.0 | 88.3 | 68.0 | 26.3 | 76.1 | 104 | 98 | 76 | 72 |
| 44 | 88.7 | 84.7 | 81.0 | 81.7 | 57.7 | 59.7 | 75.6 | 96 | 92 | 65 | 99 |
| 45 | 86.0 | 82.7 | 77.0 | 76.3 | 40.0 | 61.5 | 70.6 | 97 | 88 | 47 | 96 |
| 46 | 96.7 | 93.3 | 89.7 | 89.7 | 58.3 | 65.0 | 82.1 | 96 | 93 | 60 | 103 |
| 47 | 95.7 | 92.3 | 94.3 | 87.7 | 77.7 | 81.7 | 88.2 | 96 | 92 | 81 | 104 |
| 48 | 96.3 | 97.0 | 86.7 | 94.0 | 92.0 | 91.7 | 92.9 | 101 | 98 | 96 | 95 |

C1–C6: equilibrium scores (0 = fall, 100 = perfect stability). CDP comp: weighted composite across all six conditions. SOM: somatosensory ratio (C2/C1); VIS: visual ratio (C4/C1); VEST: vestibular ratio (C5/C1); PREF: visual preference ([C3+C6]/[C2+C5]). — denotes missing data.
