# Supplementary material for: Retained primitive reflexes are independently associated with impaired postural control in adolescents with idiopathic scoliosis
Source: Front Neurol. 2026 Sep 14;17:1921169. doi: 10.3389/fneur.2026.1921169 (PMC13616718; doi:10.3389/fneur.2026.1921169)
Supplement: Supplementary file 2 [file Table_2.DOCX]

Complete partial correlation matrix: reflexes $\times$ CDP conditions and SOT ratios, controlling for age and Cobb angle.

| **Reflex** | **Measure** | $r_{p}$ |  |  |  |
| --- | --- | --- | --- | --- | --- |
| STNR | C1 | 0.193 | nan | nan | 0.1946 |
| STNR | C2 | 0.251 | nan | nan | 0.0886 |
| STNR | C3 | 0.233 | nan | nan | 0.1154 |
| STNR | C4 | 0.105 | nan | nan | 0.4819 |
| STNR | C5 | 0.105 | nan | nan | 0.4843 |
| STNR | C6 | -0.213 | nan | nan | 0.1559 |
| STNR | CDP composite | 0.062 | nan | nan | 0.6810 |
| ATNR | C1 | -0.083 | -0.395 | 0.151 | 0.5813 |
| ATNR | C2 | -0.083 | -0.462 | 0.212 | 0.5813 |
| ATNR | C3 | -0.098 | -0.451 | 0.185 | 0.5127 |
| ATNR | C4 | -0.144 | -0.496 | 0.176 | 0.3340 |
| ATNR | C5 | -0.113 | -0.369 | 0.122 | 0.4509 |
| ATNR | C6 | -0.024 | -0.318 | 0.241 | 0.8759 |
| ATNR | CDP composite | -0.117 | -0.394 | 0.134 | 0.4318 |
| Galant | C1 | -0.048 | -0.347 | 0.227 | 0.7501 |
| Galant | C2 | -0.185 | -0.476 | 0.085 | 0.2132 |
| Galant | C3 | -0.205 | -0.462 | 0.052 | 0.1668 |
| **Galant** | **C4** | **-0.360** | **-0.612** | **-0.053** | **0.0129** |
| Galant | C5 | -0.252 | -0.501 | 0.041 | 0.0869 |
| Galant | C6 | -0.244 | -0.508 | 0.038 | 0.1029 |
| **Galant** | **CDP composite** | **-0.350** | **-0.601** | **-0.081** | **0.0159** |
| Reflex total (ATNR+Galant) | C1 | -0.072 | -0.373 | 0.199 | 0.6300 |
| Reflex total (ATNR+Galant) | C2 | -0.178 | -0.536 | 0.151 | 0.2311 |
| Reflex total (ATNR+Galant) | C3 | -0.200 | -0.471 | 0.058 | 0.1774 |
| **Reflex total (ATNR+Galant)** | **C4** | **-0.340** | **-0.594** | **-0.082** | **0.0195** |
| Reflex total (ATNR+Galant) | C5 | -0.243 | -0.488 | 0.026 | 0.0998 |
| Reflex total (ATNR+Galant) | C6 | -0.196 | -0.487 | 0.087 | 0.1912 |
| **Reflex total (ATNR+Galant)** | **CDP composite** | **-0.320** | **-0.572** | **-0.053** | **0.0282** |
| **Panel B: SOT Sensory Ratios** | | | | | |
| STNR | SOT SOM | -0.016 | nan | nan | 0.9135 |
| STNR | SOT VIS | 0.039 | nan | nan | 0.7956 |
| STNR | SOT VEST | 0.081 | nan | nan | 0.5884 |
| STNR | SOT PREF | -0.236 | nan | nan | 0.1107 |
| ATNR | SOT SOM | 0.047 | -0.202 | 0.263 | 0.7550 |
| ATNR | SOT VIS | -0.121 | -0.486 | 0.238 | 0.4164 |
| ATNR | SOT VEST | -0.062 | -0.309 | 0.179 | 0.6766 |
| ATNR | SOT PREF | 0.006 | -0.333 | 0.328 | 0.9664 |
| Galant | SOT SOM | -0.107 | -0.343 | 0.106 | 0.4726 |
| **Galant** | **SOT VIS** | **-0.341** | **-0.578** | **-0.076** | **0.0191** |
| Galant | SOT VEST | -0.223 | -0.474 | 0.095 | 0.1328 |
| Galant | SOT PREF | 0.012 | -0.280 | 0.325 | 0.9349 |
| Reflex total (ATNR+Galant) | SOT SOM | -0.063 | -0.299 | 0.126 | 0.6746 |
| **Reflex total (ATNR+Galant)** | **SOT VIS** | **-0.315** | **-0.539** | **-0.075** | **0.0311** |
| Reflex total (ATNR+Galant) | SOT VEST | -0.198 | -0.472 | 0.081 | 0.1811 |
| Reflex total (ATNR+Galant) | SOT PREF | 0.012 | -0.318 | 0.332 | 0.9354 |

Bold rows indicate associations whose 95% bootstrap confidence interval (2,000 resamples) excludes zero. All partial correlations control for age and Cobb angle via residualization. Reflex total = mean of ATNR and Galant scores (STNR excluded). SOT, Sensory Organization Test; SOM, somatosensory ratio (C2/C1); VIS, visual ratio (C4/C1); VEST, vestibular ratio (C5/C1); PREF, visual preference ratio ([C3 + C6] / [C2 + C5]). — denotes non-estimable values (typically due to near-zero variance in STNR).
