# Supplementary material for: Retained primitive reflexes are independently associated with impaired postural control in adolescents with idiopathic scoliosis
Source: Front Neurol. 2026 Sep 14;17:1921169. doi: 10.3389/fneur.2026.1921169 (PMC13616718; doi:10.3389/fneur.2026.1921169)
Supplement: Supplementary file 3 [file Table_3.DOCX]

Patient-level data: demographics, radiographic characteristics, and primitive reflex scores (Panel A).

| **ID** | **Sex** | **Age** | **BMI** | **Cobb** | **Risser** | **STNR** | **ATNR** | **Galant** | **Reflex** |
| --- | --- | --- | --- | --- | --- | --- | --- | --- | --- |
|  |  | (yr) | (kg/m${}^{2}$) | (°) |  |  |  |  | **total** |
| 1 | F | 12.0 | — | 16.3 | 6 | — | — | — | — |
| 2 | F | 10.0 | 14.1 | 12.7 | 1 | 0.00 | 0.00 | 0.50 | 0.25 |
| 3 | F | 12.0 | 13.9 | 10.0 | 7 | 0.00 | 0.50 | 0.50 | 0.50 |
| 4 | F | 10.0 | 13.3 | 10.0 | 2 | 0.00 | 0.00 | 0.00 | 0.00 |
| 5 | M | 11.0 | 17.6 | 10.9 | 1 | 0.00 | 0.50 | 1.50 | 1.00 |
| 6 | F | 11.0 | 13.3 | 19.2 | 0 | 0.50 | 0.00 | 0.00 | 0.00 |
| 7 | F | 12.0 | 16.8 | 10.3 | 7 | 0.00 | 0.50 | 0.50 | 0.50 |
| 8 | F | 12.0 | 20.3 | 12.1 | 5 | 0.00 | 0.50 | 1.00 | 0.75 |
| 9 | F | 11.0 | 20.3 | 10.4 | 6 | 0.00 | 0.50 | 0.00 | 0.25 |
| 10 | F | 11.0 | 18.4 | 18.1 | 3 | 0.00 | 0.50 | 0.50 | 0.50 |
| 11 | F | 11.0 | 20.4 | 10.5 | 2 | 0.00 | 0.50 | 0.50 | 0.50 |
| 12 | F | 11.0 | 19.8 | 11.3 | 6 | 0.00 | 0.50 | 0.00 | 0.25 |
| 13 | F | 11.0 | 14.6 | 18.2 | 2 | 0.00 | 0.50 | 0.50 | 0.50 |
| 14 | F | 12.0 | 17.0 | 12.1 | 7 | 0.00 | 1.00 | 0.50 | 0.75 |
| 15 | M | 16.0 | 23.8 | 34.7 | 7 | 0.00 | 0.50 | 0.50 | 0.50 |
| 16 | M | 13.0 | 16.6 | 31.9 | 5 | 0.00 | 0.50 | 2.00 | 1.25 |
| 17 | F | 11.0 | 11.4 | 14.9 | 2 | 0.00 | 1.00 | 0.50 | 0.75 |
| 18 | M | 9.0 | 14.8 | 16.0 | 1 | 0.00 | 1.00 | 1.00 | 1.00 |
| 19 | F | 12.0 | 16.8 | 28.6 | 2 | 0.00 | 0.50 | 0.50 | 0.50 |
| 20 | F | 14.0 | 15.8 | 14.8 | 7 | 0.00 | 0.50 | 1.00 | 0.75 |
| 21 | F | 12.0 | 18.2 | 12.6 | 5 | 0.00 | 0.50 | 1.00 | 0.75 |
| 22 | F | 12.0 | 23.4 | 21.3 | 7 | 0.00 | 0.00 | 0.50 | 0.25 |
| 23 | F | 13.0 | 16.2 | 24.9 | 3 | 0.00 | 0.50 | 0.50 | 0.50 |
| 24 | M | 8.0 | 12.9 | 11.1 | 0 | 0.00 | 2.00 | 2.00 | 2.00 |
| 25 | F | 11.0 | 11.7 | 17.9 | 0 | 0.00 | 0.50 | 0.50 | 0.50 |
| 26 | F | 13.0 | 13.3 | 24.8 | 2 | 0.50 | 0.00 | 0.00 | 0.00 |
| 27 | F | 8.0 | 13.3 | 10.9 | 2 | 0.00 | 0.50 | 0.50 | 0.50 |
| 28 | F | 13.0 | 23.1 | 18.9 | 7 | 0.00 | 0.50 | 0.50 | 0.50 |
| 29 | F | 12.0 | 15.2 | 17.1 | 7 | 0.00 | 0.50 | 2.00 | 1.25 |
| 30 | F | 13.0 | 18.1 | 14.8 | 5 | 0.00 | 0.50 | 0.00 | 0.25 |
| 31 | M | 14.0 | 18.4 | 27.8 | 4 | 0.00 | 0.50 | 2.00 | 1.25 |
| 32 | F | 12.0 | 20.1 | 22.6 | 4 | 0.00 | 0.50 | 0.00 | 0.25 |
| 33 | M | 11.0 | 15.3 | 11.1 | 0 | 0.00 | 0.50 | 1.50 | 1.00 |
| 34 | M | 9.0 | 17.4 | 12.2 | 0 | 0.00 | 0.00 | 1.50 | 0.75 |
| 35 | F | 14.0 | 14.9 | 15.2 | 7 | 0.00 | 0.00 | 0.50 | 0.25 |
| 36 | F | 10.0 | 16.7 | 10.5 | 2 | 0.00 | 0.50 | 1.00 | 0.75 |
| 37 | F | 10.0 | 16.2 | 13.2 | 2 | 0.00 | 0.50 | 1.00 | 0.75 |
| 38 | F | 11.0 | 18.0 | 10.9 | 4 | 0.00 | 0.50 | 0.00 | 0.25 |
| 39 | F | 11.0 | 14.5 | 10.9 | 0 | 0.00 | 0.50 | 0.50 | 0.50 |
| 40 | M | 9.0 | 16.5 | 14.0 | 2 | 0.50 | 0.50 | 0.50 | 0.50 |
| 41 | F | 8.0 | 12.7 | 15.0 | 1 | 0.50 | 0.50 | 1.00 | 0.75 |
| 42 | M | 14.0 | 21.5 | 10.0 | 8 | 0.00 | 0.50 | 0.00 | 0.25 |
| 43 | F | 16.0 | 18.4 | 17.0 | 8 | 0.00 | 0.00 | 0.00 | 0.00 |
| 44 | M | 9.0 | 14.7 | 16.0 | 1 | 0.00 | 0.50 | 2.00 | 1.25 |
| 45 | M | 9.0 | 17.9 | 14.0 | 2 | 0.00 | 0.50 | 0.50 | 0.50 |
| 46 | F | 13.0 | 20.5 | 19.0 | 7 | 0.00 | 0.50 | 0.00 | 0.25 |
| 47 | F | 13.0 | 20.8 | 18.0 | 7 | 0.00 | 0.00 | 0.00 | 0.00 |
| 48 | F | 14.0 | 16.2 | 21.0 | 8 | 0.00 | 0.00 | 0.00 | 0.00 |

Reflex scores are reported as the mean of left- and right-side scores (each side rated 0–4 per Pecuch et al. 2021: 0 = absent/fully integrated; 1 = low; 2 = medium; 3 = high; 4 = maximum). STNR shown for transparency but excluded from reflex total (reflex total = mean of ATNR and Galant only). — denotes missing data.
